# Assessment of UL56 Mutations before Letermovir Therapy in Refractory Cytomegalovirus Transplant Recipients

**DOI:** 10.1128/spectrum.00191-22

**Published:** 2022-03-28

**Authors:** Marta Santos Bravo, Valentin Tilloy, Nicolas Plault, Sonsoles Sánchez Palomino, María Mar Mosquera, Mireia Navarro Gabriel, Francesc Fernández Avilés, María Suárez Lledó, Montserrat Rovira, Asunción Moreno, Laura Linares, Marta Bodro, Sébastien Hantz, Sophie Alain, María Ángeles Marcos

**Affiliations:** a Microbiology Department, Hospital Clínic I Provincial de Barcelona, University of Barcelona Institute for Global Health (ISGlobal), Barcelona, Spain; b National Reference Center for Herpesviruses, Microbiology Department, CHU Limoges, Limoges, France; c UMR Inserm 1092, University of Limoges, Limoges, France; d AIDS Research Group, Institut D’Investigacions Biomèdiques August Pi I Sunyer (IDIBAPS), Hospital Clínic I Provincial de Barcelona, University of Barcelona, Barcelona, Spain; e Bone Marrow Transplant Unit, Hematology Department, Hospital Clínic I Provincial de Barcelona, Barcelona, Spain; f Infectious Diseases Department, Hospital Clínic I Provincial de Barcelona, Barcelona, Spain; University of Wisconsin-Madison

**Keywords:** cytomegalovirus, letermovir, baseline mutations, transplant recipients, phenotype

## Abstract

*De novo* mutations in the *UL56* terminase subunit and its associated phenotypes were studied in the context of cytomegalovirus (CMV) transplant recipients clinically resistant to DNA-polymerase inhibitors, naive to letermovir. R246C was the only *UL56* variant detected by standard and deep sequencing, located within the letermovir-resistance-associated region (residues 230–370). R246C emerged in 2/80 transplant recipients (1 hematopoietic and 1 heart) since first cytomegalovirus replication and responded transiently to various alternative antiviral treatments *in vivo*. Recombinant phenotyping showed R246C conferred an advanced viral fitness and was sensitive to ganciclovir, cidofovir, foscarnet, maribavir, and letermovir. These results demonstrate a low rate (2.5%) of natural occurring polymorphisms within the letermovir-resistant-associated region before its administration. Identification of high replicative capacity variants in patients not responding to treatment or experiencing relapses could be helpful to guide further therapy and dosing of antiviral molecules.

**IMPORTANCE** We provide comprehensive data on the clinical correlates of both CMV genotypic follow-up by standard and deep sequencing and the clinical outcomes, as well as recombinant phenotypic results of this novel mutation. Our study emphasizes that the clinical follow-up in combination with genotypic and phenotypic studies is essential for the assessment and optimization of patients experiencing HCMV relapses or not responding to antiviral therapy. This information may be important for other researchers and clinicians working in the field to improve the care of transplant patients since drug-resistant CMV infections are an important emerging problem even with the new antiviral development.

## INTRODUCTION

Human cytomegalovirus (HCMV) is one of the most prevalent infections in solid organ transplant (SOT) and hematopoietic stem cell transplant (HSCT) recipients. The morbidity and mortality caused by HCMV have been reduced over the last decades thanks to the development of antiviral agents and different strategies to prevent HCMV infection (1). Currently approved DNA polymerase inhibitors (valganciclovir/ganciclovir [VGCV/GCV], cidofovir [CDV], foscarnet [FOS]), are associated with significant toxicities and the emergence of drug resistance (2). The new anti-HCMV drug, letermovir (LMV), targets the terminase complex and has not presented cross-resistance with DNA polymerase inhibitors (3).

Mutations conferring resistance to LMV consist of amino acid substitutions located mainly in *UL56*, and rarely in *UL89* and *UL51* terminase subunits (4). They have been described when LMV was administered as salvage therapy of refractory or drug-resistant HCMV infections, and when used as primary or secondary prophylaxis (5, 6). It has been demonstrated that LMV resistance mutations selected *in vitro* appear earlier than for the currently used antiviral drugs (4), suggesting a lower genetic barrier of resistance to LMV. However, there is little information on whether LMV resistance mutations appeared in clinical samples prior or subsequent to LMV administration.

We aimed to determine baseline mutations in *UL56* and its associated phenotypes in the clinical context of HMCV infected SOT and HSCT recipients clinically resistant to the DNA polymerase inhibitors naive of LMV.

## RESULTS

### Genotypic antiviral resistance testing results.

HCMV *UL56* standard sequencing was performed in 80 clinical samples (75 plasma, 2 whole blood, 2 rectal biopsies, 1 aqueous humor) to detect baseline mutations comprising amino acids substitutions before LMV therapy. All samples were from refractory HCMV-infected transplant recipients (30 HSCT; 50 SOT) with resistance suspicion to DNA polymerase inhibitors.

The novel nonsynonymous point mutation R246C was detected in 2 (2.5%) transplant recipients (1 HSCT and 1 heart transplant). R246C is located in the region of *UL56* associated with resistance to LMV (aa 230–370), specifically within the leucine zipper (Fig. S1 in the supplemental material). No nucleotide changes were found in the *UL56* of the remaining patients by standard sequencing.

The *UL97* phosphokinase and *UL54* DNA polymerase were sequenced in all samples to test for mutations associated with resistance to the DNA polymerase inhibitors.

Mutations associated with antiviral drug resistance were detected in 21/80 (26.3%) patients: 18 in *UL97* (M460I/V, C480F, C592G, A594P/V, L595S/W) (7, 8), 2 in *UL54* (T700A, V781I) (9, 10), and 1 in *UL97* (M460I) in combination with *UL54* (D413N) (11). None of these subjects coincided with the 2 patients with the *UL56* R246C variant.

### Clinical follow-up of cases with the novel R246C mutation in *UL56*.

**FIG 1:**
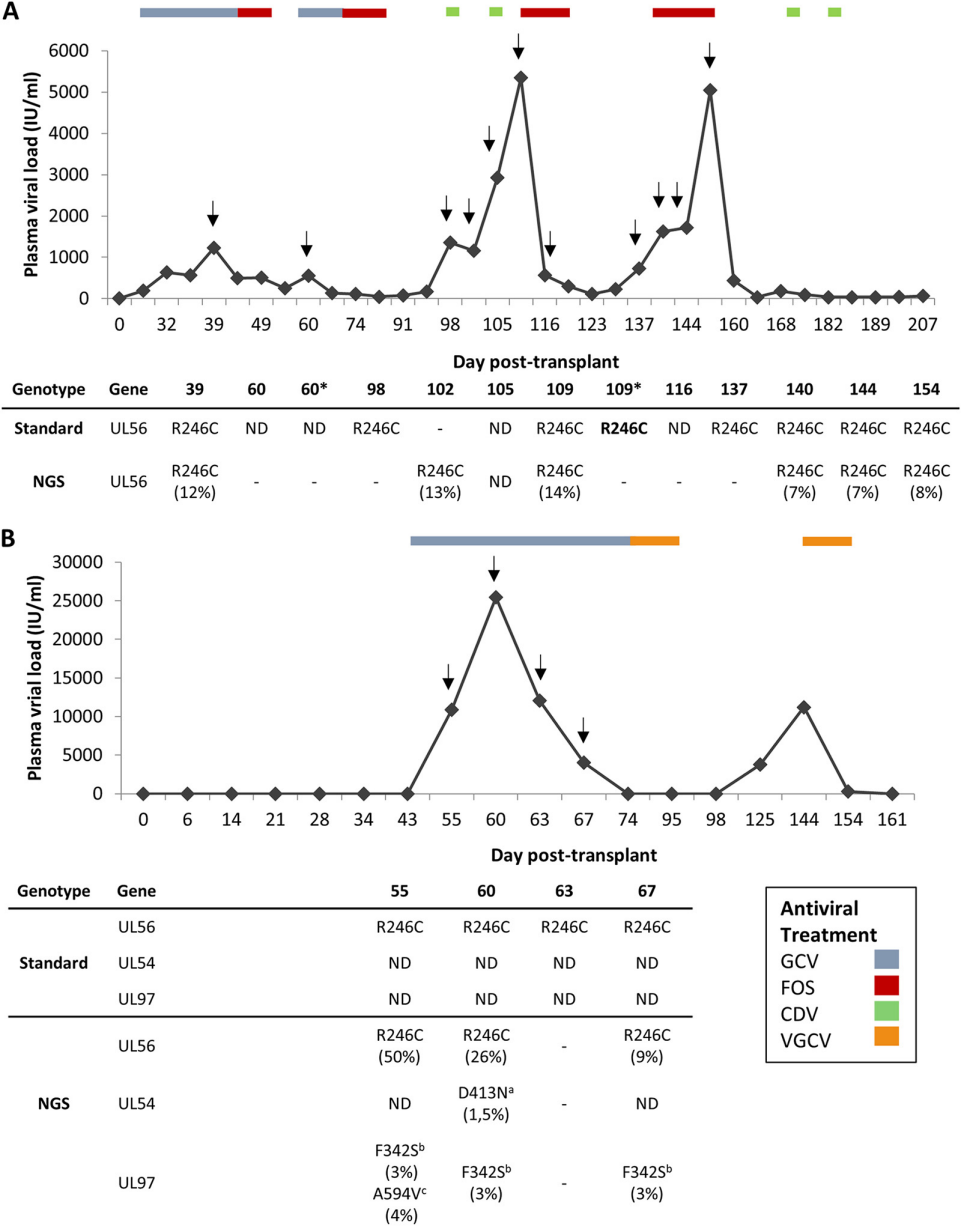
Clinical follow-up of patient 1 (A) and patient 2 (B). Viral loads (IU/mL) were tracked against days after transplantation. Antiviral treatment during follow-up is indicated in the legend, and arrows indicate the samples that were sequenced. The detection of *UL56* R246C and known antiviral-associated resistance mutations are indicated in the table below, with the allelic frequency percentages in brackets. Gastrointestinal biopsies are indicated with an asterisk. Populations 100% mutant are marked in bold. Hyphen (−) indicates that sequencing over the limit of quality could not be achieved. Abbreviations: GCV, ganciclovir; VGCV, valganciclovir; FOS, foscarnet; CDV, cidofovir; ND: no mutation detected; NGS: next generation sequencing. ^a^D413N mutation confers 10-fold-resistance to GCV, 3.8-fold shift to CDV, and sensitive to FOS (7). ^b^F342S mutation confers 8-fold resistance to GCV, 2-fold cross-resistance to MBV (26). ^c^A594V mutation confers 4.5–10.4-fold resistant to GCV (27).

Patient 1 was a 60-year-old female with chronic myelomonocytic leukemia who underwent D^-^R^+^ allogeneic HSCT from a mismatch-unrelated donor in the HCB (Fig. 1A). The conditioning regimen was based on fludarabine and busulfan for 3 days, followed by cyclophosphamide and tacrolimus as graft-versus-host-diseases (GVHD) prophylaxis.

The patient presented 4 episodes of HCMV infection. (i) At day 26 post-transplant, HCMV caused primary gastrointestinal (GI) infection which was treated with GCV and then switched to FOS due to myelotoxicity for 10 days each. (ii) 60 days after transplant, HCMV infected mainly the GI tract (biopsy viral load 2437494 copies/10^5^ cells) and was treated with GCV for 14 days but changed to FOS during 24 days until CMV-DNA clearance. (iii) 99 days after transplant, HCMV reactivation was treated with CDV every 7 days (day 99 and 106 post-transplant) and was switched to FOS for 13 days due to an increase in CMV replication in plasma (5340 IU/mL) and the GI tract (35152 copies/10^5^cells). (iv) At 137 days after transplant, the last HCMV reactivation was treated with FOS. Resistance mutation genotyping was requested and was negative for *UL97* and *UL54*, but the unknown R246C mutation in the *UL56* was detected. FOS therapy was finished when HCMV infection was resolved, and CDV was administered as maintenance treatment (2 doses 14 days apart). Unfortunately, 314 days after receiving the graft the patient died from sepsis and multiorgan failure.

All samples with viral loads >500 IU/mL from patient 1 were retrospectively genotyped for the target genes *UL56*, *UL97*, and *UL54* by standard and NGS methods (Fig. 1A). The allelic frequency of R246C was underestimated by NGS, as the depth per position reached was <1,000 reads; however, it could be clearly confirmed by the peaks observed in the Sanger sequencing, except in the GI biopsy collected on day 109 post-transplant (only the R246C variant was presented). Two naturally occurring polymorphisms (F669L, S685N) (12, 13) were detected in the *UL54* by standard sequencing and NGS, but none was identified in the *UL97* during the complete HCMV infection course.

Patient 2 was a 45-year-old man who received a D^+^R^–^ heart transplant in the HCB (Fig. 1B). Maintenance treatment consisted of cyclosporine, mycophenolate, and basiliximab. This patient did not receive prophylactic HCMV treatment, but HCMV viral loads were monitored every 15 days. This patient presented 2 HCMV infection episodes. (i) 55 days post-transplant, HCMV infection appeared and was treated with GCV for 21 days until CMV DNA clearance. During the second week of GCV treatment, viral loads rose (25,448 IU/mL) and genotype testing was requested. Standard sequencing showed the detection of the unknown *UL56* R246C variant, but no resistance mutation was found in either *UL54* or *UL97.* After DNA clearance, VGCV (450 mg/12 h) was administered for 17 days as secondary prophylaxis. (ii) 125 days post-transplant, asymptomatic HCMV reactivation in plasma was treated with VGCV (900 mg/12 h) for 10 days, resulting in CMV DNA clearance and good health controlled annually.

All the clinical isolates from the first episode were retrospectively genotyped. The *de novo UL56* R246C genetic variant was detected by standard sequencing and NGS. GCV-resistant mutations *UL97* F342S (14) and A594V (15), and minor GCV-CDV cross-resistant *UL54* D413N (11) subpopulations, were detected only by NGS. Four naturally occurring drug-susceptible polymorphisms (L655S, S685N, T885A, D898N) (12, 13, 16) were detected in *UL54* by standard sequencing and NGS, but there were no variants in *UL97*. Unfortunately, samples from the second HCMV relapse could not be recovered.

### Phenotypic study of the novel R246C mutation.

The inhibitory concentration 50% (IC_50_) value for the recombinant virus with the novel R246C mutation was compared with the AD169 control strain for GCV, CDV, FOS, maribavir, and LMV (Table 1). The resistance index (RI) was calculated as the IC_50_ of the mutant divided by the IC_50_ of the wild type AD169 strain. The mutant is considered resistant to an antiviral when RI ≥3. Results showed R246C was sensitive to GCV, CDV, FOS, MBV, and LMV.

**TABLE 1:** Antiviral susceptibility assay results of R246C

Mutation	IC_50_	Ganciclovir	Cidofovir	Foscarnet	Maribavir	Letermovir
R246C	IC_50_^*a*^	3 (± 2.0)	1.4 (± 1.8)	211.7 (± 206.4)	0.1	1.2 (± 0.8)
AD169	IC_50_^*a*^	5.2 (± 2.4)	0.8 (± 0.47)	238.3 (± 179.7)	0.2	1.5 (± 1.7)
	RI^*b*^	0.9 (± 0.9)	1.5 (± 0.9)	0.8 (± 0.3)	0.5	1.2 (± 0.5)

a IC_50_ value is the inhibitory concentration 50% (μM). Results are shown as the mean of 3 independent experiments when ganciclovir, cidofovir, and foscarnet were tested, 1 for maribavir, and 4 for letermovir and its correspondent standard deviation.

b The Resistance Index (RI) is the IC_50_ for the R246C divided by the IC_50_ of the wild-type AD169. Mutants with RI ≥3 are considered resistant.

**FIG 2:**
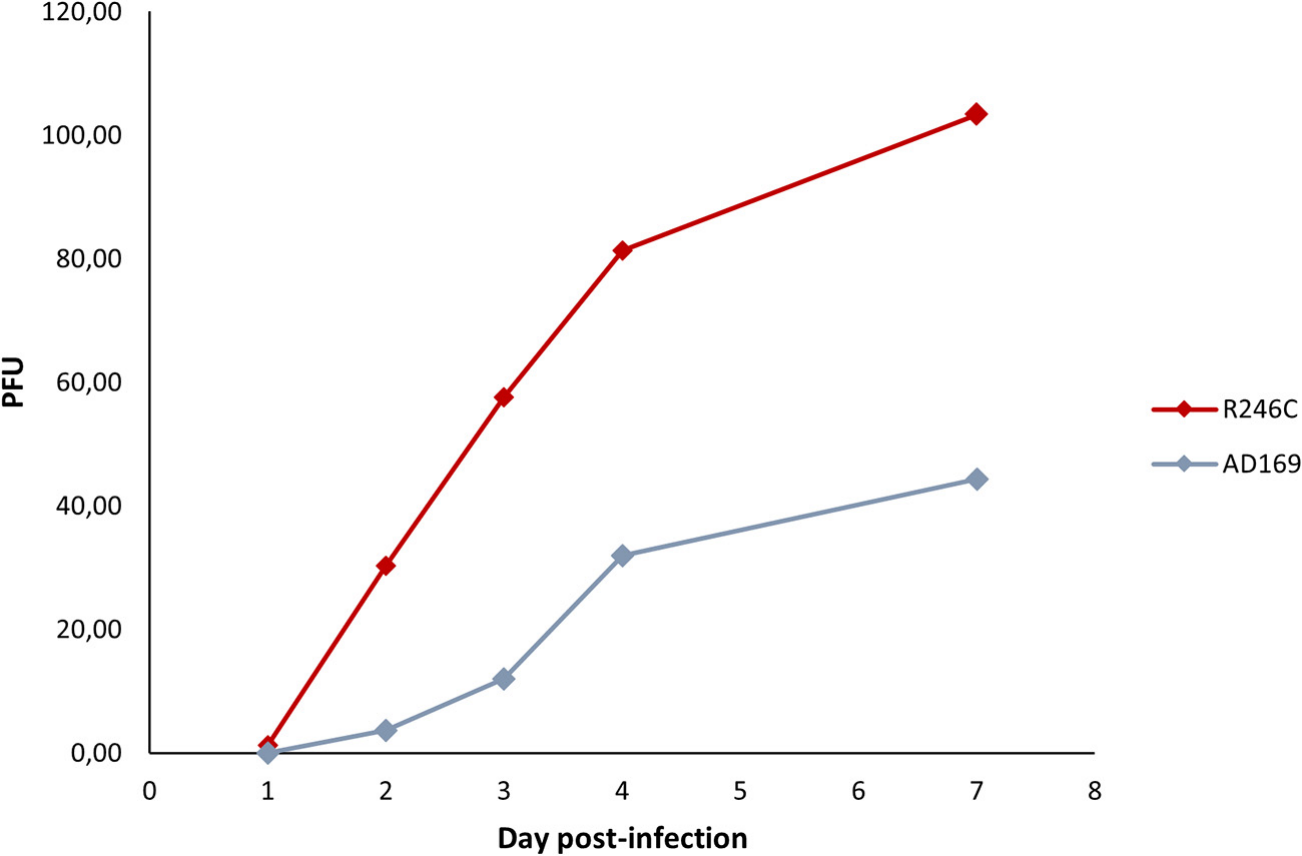
Replicative capacity assay of the R246C recombinant cytomegalovirus (CMV) strain compared with the AD169 CMV control strain. Both strains were inoculated at an equal multiplicity of infection of 0.01. PFU were counted from days 1 to 7 postinoculation. Data shown are the means of 4 independent experiments with 3 replicates per experiment.

The growth assay showed a higher replication of the R246C variant compared with the AD169, demonstrating an advantage in viral fitness with respect to the wild-type HCMV (Fig. 2).

## DISCUSSION

This study demonstrates the low frequency of genetic variants found in the *UL56* region associated with LMV resistance before LMV administration in refractory HCMV transplant recipients. The only baseline R246C mutation detected in 2/80 (2.5%) recipients (1 HSCT and 1 heart) was genetic and phenotypic studied resulting in high viral replicative capacity and a sensitive response to antiviral therapies.

Previous reports suggested that natural polymorphisms associated with standard or reduced LMV sensitivity were very unlikely in clinical isolates of patients naive of antiviral treatment (17) or treated with GCV (18), with no cross-resistance being found. Only one study reported the *de novo* LMV-resistant mutation C325Y in 2 formalin-embedded tissue biopsies from 1/147 (0.68%) patient (19).

In our two clinical contexts, the R246C variant emerged at first HCMV replication under GCV and in patient 1 reemerged every time the antiviral therapy was discontinued thanks to its advantaged growth capacity. Follow-up of the antiviral treatment and HCMV viral load monitoring of both clinical cases correlated with the antiviral-susceptibility and growth assays results, suggesting a similar impact of this mutation *in vivo.* This information is important as it has an impact in the patient management. In fact, clinical experience has shown LMV was not advisable to treat HCMV infection, due to the rapid and frequent emergence of LMV-resistant mutations once HCMV is replicating (5).

Mutations associated with resistance to LMV were found in the vicinity of R246C (V236M, T244K/R, L257I, C325Y/F/W) (20–22) in clinical isolates from HSCT and SOT recipients, comprising regular or impaired viral fitness. It is unusual to find a susceptible genetic variant within the LMV-resistant-mutation region of *UL56*, which is highly conserved among HCMV isolates, and it is even more infrequent to detect a mutation that entails a superior replicative capacity located within this polemical domain. This fact suggests that the 246 residue enhances *UL56* terminase subunit function but does not interact with LMV itself. Its position also confirms the phenomenon of vicinity of resistance and natural polymorphisms in *UL56*, already described for *UL97* phosphokinase, corroborating the suggestion that mutation phenotypes cannot be fully predicted by their gene position. Phenotyping is critically important not only to determine the resistance level to the different antiviral drugs, but also to understand its impact on the viral kinetic.

NGS is essential for correct follow-up of HCMV-complicated patients, as it enables the detection of small HCMV subpopulations that cannot be detected by standard sequencing. In our study, NGS allowed the detection and determination of the prevalence of minor *UL97* F342S, A594A, and *UL54* D413N variants in patient 2. Although assessment of the significance of minority subpopulations when using NGS in clinical samples should include evidence of their reproducible detection to avoid false positive results (23), NGS could not be repeated in our clinical cases due to the lack of sample volume. Since the evolution of resistance is staggered and progressive, monitorization for an early detection of novel genetic variants is crucial to optimized antiviral and immunosuppressive treatments and avoiding the development of resistance, which are associated with the time of exposure to the antiviral drug (15).

Overall, baseline *UL56* genetic variations before LMV therapy were infrequent in HSCT and SOT recipients. The only *UL56* R246C variant detected in this study by standard sequencing and NGS emerged in two transplant recipients since first HCMV replication and responded transiently to various alternative DNA polymerase inhibitors. Its increased growth capacity allowed its replication when therapy was discontinued. Identification of high replicative capacity variants in patients not responding to treatment or experiencing relapses could be helpful to guide further therapy and dosing of antiviral molecules.

## MATERIALS AND METHODS

### Study population.

Clinical isolates from HCMV-infected transplant recipients who filled the resistance suspicion criteria to conventional antiviral treatment (VGCV/GCV, CDV, FOS) (2) were included from April 2012 until September 2021. Antiviral treatment was administered according to the clinical judgement of the correspondent physician following the Consensus Guidelines on the Management of Cytomegalovirus in Transplantation (2). None of the patients had received LMV before sample collection. The patients were from the hospitals included in the Spanish Network for Research in Infectious Diseases (REIPI) and the Group for the Study of Infection in Transplantation (GESITRA). All samples were frozen at −80°C and sent to the Hospital Clinic of Barcelona (HCB), Spain, where genotypic antiviral resistance testing was performed.

### Genotypic antiviral resistance testing.

DNA extraction and HCMV viral load quantification was performed as described elsewhere (24).

**(i) Standard sequencing.** Genotypic testing was performed by Sanger sequencing based on PCR amplification of the HCMV *UL56* subunit of the terminase complex (residues 180–395), *UL97* phosphotransferase (400–670) and *UL54* DNA polymerase (300–1,000) regions corresponding to resistance-associated domains using the primers and procedure described previously (25). Each isolate was bidirectionally sequenced 3 times to avoid artifacts.

For patients with *UL56* genetic variants, samples with HCMV viral loads >500 IU/mL were retrospectively sequenced by standard and next generation sequencing (NGS) for *UL54*, *UL56*, and *UL97* to determine the timing of the earliest emergence of the respective mutations.

**(ii) Next generation sequencing (NGS).** A set of 100 strain sequences was provided to the Paragon Genomics teams (CA, USA) for custom panel design of full-length *UL54*, *UL56*, and *UL97* genes in HCMV. A whole-genome alignment of all 100 strains was performed, and this aligned sequence was used to generate a two-pool primer design using the Paragon Genomic's in-house primer design pipeline, ParagonDesigner. The design was able to achieve 100% coverage of all the regions of interest using 267 primers for a total of 57 amplicons. The design incorporated a small number of degenerate primers to account for potential single nucleotide polymorphisms in priming regions. The primers were synthesized with Illumina-specific partial adapters and provided by Paragon Genomics together with Targeted Library Kit Reagents and accessories (CleanPlex Custom Panel, SKU 916028).

Libraries were quantified using the QuantiFlour ONE dsDNA System (Promega, USA) and Bioanalyser (Agilent Technologies) and normalized to 4 nM final concentration. Samples were sequenced using the MiSeq platform (Illumina, USA). Bioinformatics analysis was performed at the French Reference Center for Herpesviruses Genomic Platform (CHU Limoges). Quality check of raw-reads was performed using FastQC v.0.11.5 and MultiQC v.0.9. A trimming step using Trimmomatic-0.39 was done using low stringency parameters (phred33; LEADING:3; TRAILING:3; SLIDINGWINDOW:4:15; MINLEN:36). Trimmed reads were then validated using the previous quality check step. Trimmed reads were aligned against the AD169 reference strain (GenBank: X17403.1) using the BWA-MEM algorithm from Bwa 0.7.17-r1188 (seed 20) and Samtools 1.9 (view, sort, and index options). Genetic variations from alignment were called using Lofreq 2.1.3.1, and variant annotations were made with SnpEff 4.3 software. Mutations were then filtered using Bcftools 1.8 based on Phred score (200) and depth (100). Variant call format (VCF) files were then formatted using SnpSift 4.3.1t (extractFields) and custom sed commands. Consensus files were obtained from VCF files using Bcftools 1.8 (consensus) and formatted using awk custom commands.

### Phenotypic assay by recombinant bacterial artificial chromosome (BAC) technology.

*UL56* R246C mutation, with an unknown phenotype at the time of genotypic detection, was individually tested at the French National Reference Center for Herpesviruses (Limoges) using a phenotypic assay with recombinant bacterial artificial chromosomes (BAC) technology as described previously (26). R246C single mutation was introduced by “en passant” mutagenesis into a human cytomegalovirus (HCMV) BAC (27) containing an enhanced green fluorescent protein (EGFP) gene in the unique short region derived from the AD169 laboratory strain (provided by M. Messerle). The recombinant BAC was transfected into MRC-5 cells (bioMérieux, Lyon, France) using the liposomal reagent Transfast (Promega, Madison, Wisconsin) following the manufacturer’s instructions. The presence of the mutation was confirmed by Sanger sequencing.

A focus reduction assay in a 48-well MRC-5 fibroblast culture plate with a multiplicity of infection (MOI) of 0.01 was used to assess antiviral susceptibility in triplicate to GCV, CDV, FOS, maribavir, and LMV on the basis of fluorescent cytopathic foci counted in an ECLIPSE E200 analyzer (Nikon, New York, USA). Inhibitory concentration 50% (IC_50_) of the mutant was compared to that of the wild-type HCMV BAC control.

To estimate the impact of the R246C mutation on viral fitness, the recombinant strain and the AD169-EGFP wild-type control were inoculated into 48-well MRC-5 culture plates with a MOI of 0.01. The number of fluorescent cytopathic foci was counted from days 1 to 7 postinoculation to establish viral growth curves for each recombinant.

### Ethical approval.

This study was approved by the Ethical Committee of the HCB (ref.HCB/2018/0634) as the reference committee for all the participating hospitals endorsed by GESITRA according to CPMP/ICH/135/95 regulations. Signed informed consent was obtained from all the patients included in the study.
