# Supplementary material for: Assessment of UL56 Mutations before Letermovir Therapy in Refractory Cytomegalovirus Transplant Recipients
Source: Microbiol Spectr. 2022 Mar 28;10(2):e00191-22. doi: 10.1128/spectrum.00191-22 (PMC9045154; doi:10.1128/spectrum.00191-22)
Supplement: SUPPLEMENTAL FILE 1 — Supplemental material. Download SPECTRUM00191-22_Supp_1_seq1.pdf, PDF file, 0.2 MB [file spectrum00191-22_supp_1_seq1.pdf]

SUPPLEMENTARY DATA

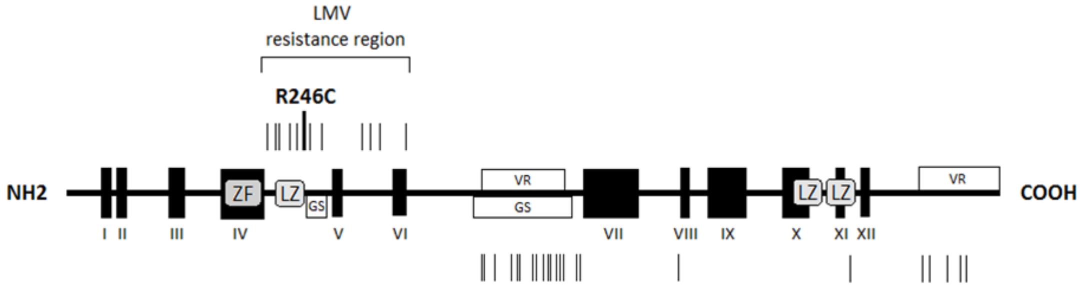

Figure S1. Scheme of the protein UL56 domain organization according to [1] with the novel R246C mutation indicated. Conserved regions are indicated in black boxes (domains I-XII); variable regions (VR) and glycine and serine-rich flexible region (GS) in white boxes; leucine zippers (LZ) and zinc finger domain (ZF), the metal-binding site of which is located in region IV. The location of letermovir-resistant mutations previously described *in vitro* are shown above [2, 3]. Letermovir-sensitive associated polymorphysims described previously are shown below [1, 4].

REFERENCES

1. Champier G, Couvreux,A, Hantz S, Rametti A, Mazeron,MC, Bouaziz S, Denis F, Alain S. Putative functional domains of human cytomegalovirus pUL56 involved in dimerization and benzimidazole D-ribonucleoside activity. Antivir Ther **2008** 13, 643e654.
2. Goldner T, Hempel C, Ruebsamen-Schaeff H, Zimmermann H, Lischka P. Geno and phenotypic characterization of human cytomegalovirus mutants selected in vitro after letermovir (AIC246) exposure. Antimicrob Agents Chemother **2014**; 58:610‐3.
3. Chou S. Rapid In Vitro Evolution of Human Cytomegalovirus UL56 Mutations That Confer Letermovir Resistance. Antimicrob Agents Chemother. **2015**; 59(10):6588-93.
4. Peter Lischka, Douglas Zhang, Daniel Holder, Holger Zimmermann. Impact of glycoprotein B genotype and naturally occurring ORF UL56 polymorphisms upon

susceptibility of clinical human cytomegalovirus isolates to letermovir. Antiviral Research **2016** 132, 204-209.
